# The Extinction of Tuberculosis

**Published:** 1903-03-21

**Authors:** 


### THE EXTINCTION OF TUBERCULOSIS.

In a paper read before the Society of Medical Officers of Health Dr. Hillier[1] deals with this important problem. He introduces the subject by giving the statistics of the tuberculosis death-rate in Prussia for the last 25 years, and comparing them with similar figures for England and Wales. The English decline in the tuberculosis death-rate during that period is a fairly constant one, the decline in Prussia is not of the same character. In England the deaths from phthisis have been declining since 1838, when they stood at 38 per 10,000, down to the present time, when they are only 18 per 10,000. This decline, in the opinion of many competent authorities, is associated with, and largely attributable to, the generally improved sanitary conditions and food standards among the working classes. Professor Koch is also of opinion that the degree of segregation and isolation afforded by our chest and general hospitals has materially aided in this fall. In Prussia, whose statistics deal, not with the phthisis rate alone, but with all deaths from tuberculosis, the only decline—and that is a rapid one—of which the statistics furnish a record, has occurred since 1886. From 1876 to 1886 the Prussian death-rate from tuberculosis remained stationary at about 31 per 10,000. In 1887 the rate began to drop and it has continued to diminish comparatively steadily down to 1900, when it had fallen to 21 per 10,000. In England in the same period the drop in the tuberculosis death-rate has only been from 24 to 19 per 10,000. To what, then, is the rapid fall in the Prussian tubrculosis death-rate due? Dr. Hillier believes it may be attributed to three main causes, apart from improved sanitation, which probably has also influenced the fall. The three causes are :—(1) The discovery of the tubercle bacillus in 1881, and the spreading of the knowledge of the infectious character of phthisis. (2) The greatly improved general condition of the working classes brought about by the operation of the Workman's State Insurance Laws, the first of which, the Sick Insurance Law, came into existence in 1883. (3) The establishment of sanatoria, although this is too recent a movement in Prussia to have had much effect at present. So far, so good. Tuberculosis is diminishing both in England and in Germany, and if it continue to do so at the present rate it will disappear from both countries in about a generation, as leprosy has disappeared from these countries before it, though at the present rate of progress it will disappear from Germany first. But Dr. Hillier does not consider that this rate of progress is good enough. More energetic steps should be taken to extinguish the disease, for even now it remains the deadliest pestilence in our midst ; and it should not be forgotten that compared to its annual death-roll in the British dominions alone, our losses in the South African war were a bagatelle ; that it is still the chief cause of domestic tragedy and widespread human misery ; that in England and Wales, the countries least affected in Northern Europe, the deaths amount to 60,000, and throughout Europe to 1,000,000 every year ; and finally it should be remembered that it is a preventable infectious disease subject to human control. Professor Koch is quoted as having stated that the last stages of phthisis are the most dangerous of all from the point of view of spreading infection, and that properly collecting and sterilising the sputum is not sufficient to prevent dissemination of the disease among those who come into contact with the patient who is suffering from advanced pulmonary tuberculosis. In Professor Koch's opinion the most potent factor in causing infection is the microbe-laden spray which spreads around him when the consumptive coughs or speaks. Numerous experiments have been made which have successfully demonstrated this tubercle-bacillus-containing spray, the most interesting being those of Flügge and Heymann, who infected healthy guinea-pigs with pulmonary tuberculosis by having them coughed at by consumptives. Having dealt fully with the question of the infectiousness of phthisis, Dr. Hillier submits in outline a scheme which he believes the present position demands. He suggests that notification of phthisis should be compulsory, that such a degree of isolation as is possible should be enforced in all cases of advanced phthisis, and that provision should be made for an adequate increase of the staffs of all medical officers of health, to be specially devoted to the supervision and control of phthisis.

[1] Public Health, March, 1903.

